# NATURALISTIC DRIVING BEHAVIOR AS A NEUROBEHAVIORAL MARKER OF PRECLINICAL ALZHEIMER’S DISEASE

**DOI:** 10.1093/geroni/igz038.3243

**Published:** 2019-11-08

**Authors:** Ganesh Babulal

**Affiliations:** Washington University School of Medicine (St. Louis), St. Louis, Missouri, United States

## Abstract

Decline in driving skills begins in preclinical AD, when an older adult remains cognitively normal, but the underlying disease process has begun. Preclinical AD is detectable among cognitively normal individuals using molecular biomarkers: positron emission tomography (PET) imaging and cerebrospinal fluid (CSF). The aim of this prospective, longitudinal study is to determine whether naturalistic driving behavior using in-vehicle dataloggers can distinguish older adults with (n=36) and without preclinical AD (n=134). Driving data was calculated as mean/month for several variables (number of trips/day, trip length, trip time, speeding, and hard-braking) for participants followed between one to 46 months. Using stepwise logistic regression, the area under the receiver operating curve (AUC) and 95% confidence interval for these five variables was 0.73 (0.63-0.79) in distinguishing those with and without preclinical AD via amyloid imaging. When age, gender, race, and education were added, the model improved: 0.80 (0.72-0.88). Finally, when apolipoprotein ε4 allele (APOε4), obtained via blood or saliva, was added to the model, accuracy improved: 0.84 (0.77-0.89). Similar results were found using CSF biomarker tau/Aβ42: AUCs (95% CI) were 0.68 (0.58-0.79) for driving variables alone, 0.77 (0.69-0.86) for driving variables and demographics, and 0.87 (0.80-0.94) driving variables, demographics, and apolipoprotein ε4 allele. These promising findings suggest that naturalistic driving behavior can predict those with and without preclinical AD. The AUC is further improved with demographics and APOε4, an easily obtainable genetic biomarker. This model may be used in clinical/research settings as a screen or adjunct for diagnostics and prognostics purposes.

